# False and true pre-treatment predictors of weight loss in obese patients starting a program for lifestyle change

**DOI:** 10.1007/s40519-014-0126-3

**Published:** 2014-05-11

**Authors:** Barbara Cresci, Laura Pala, Roberta Poggiali, Cosetta Guarnieri, Edoardo Mannucci, Michela Bigiarini, Carlo Maria Rotella

**Affiliations:** 1 Endocrinology Unit, Careggi University Hospital, Florence, Italy; 2 Dietetic Unit, Careggi University Hospital, Florence, Italy; 3 Diabetes Agency, Careggi University Hospital, Florence, Italy; 4 Department of Biomedical Experimental and Clinical Sciences and Obesity Agency, University of Florence, Viale Pieraccini 6, 50134 Florence, Italy

**Keywords:** Obesity, Outcome, Muscle mass, Weight loss

## Abstract

**Purpose:**

Weight loss treatment effectiveness and cost-effectiveness may be improved by the identification of patients who are more prone to participate and gain benefit from specific interventions. Aim of the present study is to identify easily available additional predictors of weight loss among data usually present in the medical records of obese/overweight patients attending an outpatient clinic for a non-pharmacological lifestyle change program.

**Results:**

268 patients, 74 men and 195 women (age 43.2 ± 11.9 years, BMI 38.9 ± 6.8 kg/m^2^) were enrolled. Among these patients, only 35.6 % men and 22.7 % women completed the 6-month protocol. Among participants, 50.7 % lost at least 5 % initial body weight after 6 months (SUCCESSES), while 49.3 % failed (FAILURES). Baseline nutritional parameters (total Kcal, lipid, carbohydrate, protein and alcohol intake) were not significantly different in successes when compared to failures, while a significant difference between groups was observed for baseline diastolic blood pressure (DBP); free fat mass (FFM); muscle mass (MM); total body water (TBW); HDL cholesterol; ALT; AST; γGT. After dividing into quartiles the not-normally distributed variables, successes had AST values above median (3rd and 4th quartiles; *χ* ^2^ = 0.003). At multivariate analysis (linear regression), the OR was 3.34 (1.42–7.85; *p* = 0.006).

**Conclusions:**

In our patients, baseline liver enzyme levels (AST in particular), but not baseline quantitative and qualitative dietary intake, were significantly different in successes versus failures and could therefore represent a predictor of success. In conclusion, AST could represent a usually available biomarker that could be used as a predictor of outcome (weight loss) in obese patients starting a lifestyle change program.

## Introduction

Weight loss programs for obese patients, particularly those lifestyle change-oriented and without any pharmacological treatment, are known to be time consuming and usually show low percentages of success in terms of weight loss for a relevant number of different reasons [1]. At this purpose, weight loss treatment effectiveness and cost-effectiveness may be improved, not only by the identification of factors affecting weight loss, but mainly by the identification of patients who are sufficiently motivated and thus more able to participate and gain benefit from the intervention.

Many parameters have been identified with a potential capacity of predicting success/failure in a weight loss program [2–4]. Among these, the most suggestive could be considered: self-motivation [5–7]; self-efficacy [8]; active lifestyle [9]; more relevant initial weight loss [10]; social and/or family support [11, 12].

Our group has already explored this area, reporting that therapeutic success is predicted by basal TRE-MORE test scores and by estimated muscle mass. In fact, we have found that a higher muscle mass, as estimated through bioimpedance analysis (BIA), is associated with a greater weight loss in obese patients [13]. Moreover, we demonstrated that therapeutic success is predicted by TRE-MORE test scores. In particular, TRE-MORE total score is a predictor of failure, but not of attendance, whereas drop-out patients showed a lower score only in TREMORE-3 subscale which investigates lifestyle habits [14, 15].

A substantial body of literature exists suggesting that weight loss can be achieved by varying the macronutrient distribution and composition of dietary factors. Champagne et al. [16] have investigated the effect of dietary intake modifications on weight loss and maintenance during an intensive behavioral weight loss program, concluding that success was mainly associated with increased intake of proteins, fruits and vegetables, and low-fat dairy. Moreover, many studies have explored the possibility of using the variation in the intake of different macronutrients as predictors of weight loss [17–19]. Very few studies, on the contrary, explore pre-treatment dietary habits impact on treatment success. Recently, Byrne et al. [20] evaluated the effects of pre-treatment self-efficacy for diet and exercise, as well as changes in self-efficacy occurring during treatment, on weight loss success, demonstrating that treatment attendance and changes in exercise self-efficacy during treatment were stronger predictors of weight loss than changes in diet.

It is well known that obesity is frequently associated to many other morbidities. The majority of these complications are related to comorbid conditions that include coronary artery disease, hypertension, type 2 diabetes mellitus, respiratory disorders and dyslipidemia. Therefore, patients starting a weight loss program are usually screened for biohumoral parameters related to these complications, such as lipid profile, glycemia, insulinemia, blood pressure levels and liver function, the potentiality of which as predictors has not yet been demonstrated.

Aim of the present study is to identify easily available predictors that could be used as additional predictors of weight loss among data present in the medical records of obese/overweight patients attending an outpatient clinic.

## Methods

### Patients

The study was carried out in the Outpatient Clinic of the Obesity Agency of the University of Florence. Complete baseline data including blood samples results were collected for 268 patients out of the enrolled 331 seeking treatment for overweight/obesity in our outpatient clinic [15].

Inclusion and exclusion criteria are shown in Table 1.

**Table 1:** Inclusion and exclusion criteria of patients

Inclusion criteria (A)
Obesity or overweight (BMI ≥ 27 kg/m^2^)
Age 18–65
Residence within 40 km from the Clinic
Informed consent
Exclusion criteria (B)
Patients living at distances more than 40 km
Uncontrolled endocrine disorders, such as hypo- or hyperthyroidism
Diabetes
Pregnancy
Illiteracy, or inadequate knowledge of the Italian language
Any condition interfering with the possibility of regular physical exercise (e.g., severe cardiac dysfunction, severe respiratory insufficiency, major neurologic disorders, etc.)
Diagnosis of major depression, bipolar disorder, obsessive–compulsive disorder, schizophrenia, or mental retardation
Current treatment with antipsychotics, antiepileptics, tricyclic antidepressants, or lithium
Intention to move more than 40 km far from Florence in the following 12 months

Before the collection of data, during the first routine visit, the procedures of the study were fully explained; after that, the patients were asked to provide their written informed consent to the participation to the study. The study protocol had been previously approved by the Local Ethical Committee. The treatment protocol was carried out as previously described [15].

### Measurements

At baseline, an Endocrinologist collected an accurate medical history, performed a complete physical examination and measured anthropometric parameters (BMI, waist circumference, blood pressure and heart rate). Body fat (BF) and lean body mass were estimated through BIA, performed by a tetrapolar single frequency (50 kHz) phase-sensitive impedance analyzer (QUANTUM/S; AkernSrl, Firenze, Italy). In the same day, the patients also met a dietician (R.P. and C.G.) who assessed usual food intake on the basis of a 30-day recall, using a dedicated software (Gedip by Solutions S.n.c, Italy), together with photo atlas for the determination of portions (Scotti-Bassani Institute, Italy). A goal of a 500 kcal/day reduction from usual food intake was agreed upon with the patient. To reach that objective, the patients were asked to self-monitor their food intake for at least a week before each monthly follow-up visit with the dietitian [15]. Blood samples were drawn after 12-h overnight fast for glucose (Beckman instruments, Fullerton, CA, USA), TSH (Electrochemiluminescent, Modular Roche, Milan, Italy), transaminases, gamma-GT, insulin (electrochemiluminescence immunoassay, Roche Diagnostics, Mannheim, Germany), cholesterol, HDL-cholesterol, triglycerides (for lipid panel: Abbott Bichromatic Analyser (Abbott Diagnostics, South Pasadena, CA, USA). All laboratory determinations were performed in the central Laboratory of Careggi Hospital in Florence.

Final measures, including BMI and waist, were considered those collected after 6 months from the enrollment in the protocol.

### Definition of outcomes

Therapeutic success was defined as a weight loss at 6 months of at least 5 % from baseline to assess this outcome.

### Statistical analysis

Continuous variables were reported as mean ± standard deviations (SD), whereas categorical variables were reported as median and limits of confidence. Between-group comparisons of continuous variables were performed using unpaired Student’s* t* test or Mann–Whitney *U* test, for variables with normal or non-normal distribution, respectively. Correlation analyses were performed with Spearman’s method. Logistic regression analyses were applied for dichotomous outcomes (successes vs. failures).

All analyses were performed using SPSS for Windows 15.0 (Chicago Inc, USA).

## Results

268 patients, 74 men and 195 women (age 43.2 ± 11.9 years, weight 105.6 ± 22.6 kg, BMI 38.9 ± 6.8 kg/m^2^, waist 116.1 ± 16.2 cm) were enrolled. Among these patients, only 26 (35.6 %) men and 44 (22.7 %) women completed the 6-month protocol. Among participants, 50.7 % (*n* = 70) lost at least 5 % initial body weight after 6 months (and will be thereafter called SUCCESSES), while 49.3 % (*n* = 68) failed (FAILURES).

Baseline nutritional parameters, assessed as previously described, were also analyzed (Table 2). In particular, total Kcal intake, lipid intake, carbohydrate intake, protein intake, together with baseline alcohol intake were not significantly different in successes when compared to failures. Furthermore, the spontaneous difference in total kcal intake, lipid intake, carbohydrate intake, protein intake, alcohol intake recorded after 1-week self-monitoring was not significantly different in successes when compared to failures.

**Table 2:** Baseline nutritional parameters: differences between successes and failures

Baseline intake	Successes	Failures	*p*
Kcal	2,265.1 ± 887.4	2,032.7 ± 856.1	0.121
Proteins (g)	88.3 ± 33.8	83.7 ± 31.5	0.513
Lipids (g)	82.1 (68.3–106.1)	81.0 (65.0–102)	0.784
Saturated lipids (g)	25.0 ± 10.4	22.2 ± 12.1	0.228
Carbohydrates (g)	293.8 ± 136.6	253.4 ± 118.2	0.067
Alcohol (g)	5.0 (1.2–23.5)	4.4 (1.0–19.9)	0.511

Data are shown as media ± SD, whereas categorical variables were reported as median and limits of confidence

Differences between baseline data of successes (losing at least 5 % of initial body weight at the intention-to-treat analysis) and failures (completers losing <5 % of initial body weight and drop-outs) are summarized in Table 3. A significant difference was observed only for diastolic blood pressure (DBP) (78 ± 6 vs. 75 ± 9 mmHg; *p* = 0.033); free fat mass (FFM) (62.8 ± 15.1 vs. 57.8 ± 9.3 %; *p* = 0.021); muscle mass (MM) (45.2 ± 12.6 vs. 40.6 ± 7.6 %; *p* = 0.011); total body water (TBW) (46.3 ± 11.1 vs. 42.5 ± 6.7 %; *p* = 0.016); HDL (44.9 ± 11.9 vs. 50.5 ± 14.8 mg/dl; *p* = 0.020); ALT (29.6 ± 30.8 vs. 20 ± 5 mg/dl; *p* = 0.017); AST (40.4 ± 51.2 vs. 25.5 ± 12.3 mg/dl; *p* = 0.027); γGT (38.6 ± 52.3 vs. 22.2 ± 11.4 mg/dl; *p* = 0.019).

**Table 3:** Differences between baseline data of successes (losing at least 5 % of initial body weight at the intention-to-treat analysis) and failures (completers losing <5 % of initial body weight and drop-outs)

	Successes	Failures	*p*
DBP (mmHg)	78 ± 6	75 ± 9	0.033
FFM (%)	62.8 ± 15.1	57.8 ± 9.3	0.021
MM (%)	45.2 ± 12.6	40.6 ± 7.6	0.011
TBW (%)	46.3 ± 11.1	42.5 ± 6.7 %	0.021
HDL (mg/dl)	44.9 ± 11.9	50.5 ± 14.8	0.020
ALT (mg/dl)	29.6 ± 30.8	20 ± 5	0.017
AST (mg/dl)	40.4 ± 51.2	25.5 ± 12.3	0.027
γGT (mg/dl)	38.6 ± 52.3	22.2 ± 11.4	0.019

Data are shown as media ± SD

None of these differences was retained at multivariate analysis with only one exception. In fact, after dividing into quartiles the not-normally distributed variables, it resulted that successes have AST values above median (3rd and 4th quartiles; *χ* ^2^ = 0.003), even if a trend was observed for ALT (3rd and 4th quartiles; *χ* ^2^ = 0.068) and γGT (3rd and 4th quartiles; *χ* ^2^ = 0.074). At multivariate analysis (logistic regression), after adjusting for age and waist, the OR for AST was 3.34 (1.42–7.85; *p* = 0.006).

## Discussion

The present study tries to identify new possible predictors of outcome in a non-pharmacologic lifestyle change-centered weight loss program. For this purpose, we analyzed a series of baseline blood parameters, which are usually studied in obese patients to assess the presence of eventual comorbidities. In particular, successes resulted to have GOT/AST values above median.

It is well known that obesity is frequently associated with a cluster of risk factors including dysglycemia, hypertension and dyslipidemia, in a few words defined by the concept of metabolic syndrome (MS). As a consequence, obese patients are often affected by non-alcoholic fatty liver disease (NAFLD). Impaired hepatic fatty acid (FA) turnover together with insulin resistance are key players in NAFLD pathogenesis [21]. Most individuals are asymptomatic and are usually discovered incidentally because of abnormal liver function tests. Elevated liver biochemistry is found in 50 % of patients with simple steatosis. Change in liver function tests is considered as surrogate marker of liver injury and non-alcholic fatty liver disease (NAFLD). Previous studies have demonstrated that circulating concentration of liver function tests like γ-glutamyltransferase (γGT), alanine aminotransferase (ALT) and aspartate aminotransferase (AST) is increased in individuals with insulin resistance and the metabolic syndrome [22]. In addition, these components of liver function tests have been shown to be positively associated with the risk of future type 2 diabetes [23, 24]. In particular, available data indicate moderate associations of ALT and γGT with risk of type 2 diabetes events, and no evidence for an increased risk with AST [25, 26].

Moreover, recent data [27] speculate that abnormal levels of ALT and AST are associated with a deregulation of normal amino acid metabolism in the liver, including aromatic amino acid, and then special compounds such as glutamate are released into the general circulation. This hypothesis attempts to illustrate the critical role of the “liver metabolism” in the pathogenesis of the MS and postulates that before the liver becomes fatty, abnormal levels of liver enzymes might reflect high levels of hepatic transamination of amino acids in the organ. Anyway, elevated transaminase levels could represent an early at-risk situation of pre-NAFLD.

In our patients, baseline liver enzyme levels (AST in particular), but not baseline quantitative (total Kcal) and qualitative (lipids, carbohydrates, proteins, alcohol) dietary intake, were significantly different in successes versus failures and could therefore represent a predictor of success. Successes have AST values above median. A possible explanation could be that AST is raised in acute liver damage, but is also present in red blood cells, and cardiac and skeletal muscle and is therefore not specific to the liver. For instance, in athletes, the interpretation of serum aminotransferases concentrations should consider the release of AST from muscle and of ALT mainly from the liver [28]. Therefore, we could speculate that the predictive value of AST but not of ALT or γGT in our population could be related to the significantly different amount of baseline muscle mass in the two populations. This could be consistent with our previous findings that successes have better baseline grade of fitness in terms of initial muscle mass when compared to failures [15].

Both genetic and environmental factors have been proposed to be involved in the etiology of NAFLD. Thus, nutrition is reasonably considered to be a potential environmental factor affecting the risk for this disease. Although there is consistent evidence that overweight due to energy overconsumption increases the risk for and the prevalence of fatty liver, the role of diet composition, in terms of macro- or micronutrients, in the pathogenesis of the disease remains controversial [29]. Dietary habits shown by patients before starting the lifestyle change program have not been demonstrated to be significantly different between those losing at least 5 % of initial body weight and failures. The same result was obtained comparing the basal and 1 week after quantitative and qualitative dietary intake, spontaneously modified by patients without the dietician intervention. Anyway, we should point out that both baseline total Kcal and carbohydrate intake, even if not reaching the statistical significance, show different values when comparing successes and failures. In particular, failures report lower total Kcal intake and lower carbohydrate intake before entering the program. It should be taken into consideration that data regarding qualitative and quantitative dietary intake were collected based on information self-reported by patients. The tools used by the operators, such as a dedicated software and a photo atlas representing different portion sizes, were aimed at reducing the bias, but the problem related to an altered perception from the patient’s point of view could nevertheless be still present.

Moreover, despite worldwide guidelines and recommendations, research examining diet composition for the management of obesity remains controversial. Previous studies have found that low-fat diets promote short-term weight loss [30]; however, some studies suggest that low-carbohydrate, high-protein, and high-fat diets may also result in substantial weight loss [31]. We can therefore conclude that a substantial body of literature exists suggesting that weight loss can be achieved by varying the macronutrient distribution and composition of dietary factors, even if evidence for type of diet on long-term weight maintenance remains debatable [16, 32]. On the other hand, few data are present regarding quality of eating patterns as predictors of weight loss. Hart et al. [33] demonstrated that early changes in eating habits, but not baseline eating behaviors may promote greater BMI reductions in a cohort of adolescent patients. These findings are consistent with our data, even if referred to a completely different population.

Based on our data, we can at the moment conclude that neither usual dietary habits nor self-management operated by patients based on personal beliefs could represent a good predictor of success for this kind of programs. Finally, these results confirm once again that baseline grade of fitness (i.e., initial muscle mass) is a better predictor of success, rather than dietary habits, when starting a lifestyle modification program, as already demonstrated by our group [15].

Moreover, we can conclude that AST could represent a usually available biomarker that could be used as a predictor of weight loss in obese patients starting a lifestyle change program. Nevertheless, this should be considered as a pilot study which includes a reasonable number of patients and therefore the efficacy of present data, which aim to identify a weight loss predictor of outcome, will need to be verified through specifically designed longer-term randomized clinical trials enrolling a larger number of patients.
